# Pathobiology of the highly pathogenic avian influenza viruses H7N1 and H5N8 in different chicken breeds and role of Mx 2032 G/A polymorphism in infection outcome

**DOI:** 10.1186/s13567-020-00835-4

**Published:** 2020-09-10

**Authors:** Raúl Sánchez-González, Antonio Ramis, Miquel Nofrarías, Nabil Wali, Rosa Valle, Mónica Pérez, Albert Perlas, Natàlia Majó

**Affiliations:** 1 IRTA, Centre de Recerca en Sanitat Animal (CReSA, IRTA-UAB), Campus de la Universitat Autònoma de Barcelona (UAB), Bellaterra, España; 2 Departament de Sanitat i Anatomia Animals, Universitat Autònoma de Barcelona, Campus de la Universitat Autònoma de Barcelona (UAB), Bellaterra, España

**Keywords:** highly pathogenic avian influenza, Gs/GD lineage, classical strain, chickens, breed, pathogenicity

## Abstract

Chickens are highly susceptible to highly pathogenic avian influenza viruses (HPAIVs). However, the severity of infection varies depending of the viral strain and the genetic background of the host. In this study, we evaluated the pathogenesis of two HPAIVs (H7N1 and H5N8) and assessed the susceptibility to the infection of local and commercial chicken breeds from Spain. Eight chicken breeds were intranasally inoculated with 10^5^ ELD_50_ of A/Chicken/Italy/5093/1999 (H7N1) or A/Goose/Spain/IA17CR02699/2017 (H5N8 clade 2.3.4.4. B) and monitored during 10 days. Chickens were highly susceptible to both HPAIVs, but H7N1 was considerably more virulent than H5N8 as demonstrated by the highest mortality rates and shortest mean death times (MDT). Both HPAIVs produced severe necrosis and intense viral replication in the central nervous system, heart and pancreas; however, the lesions and replication in other tissues were virus-dependent. High levels of viral RNA were detected by the oral route with both viruses. In contrast, a low number of H5N8-inoculated chickens shed by the cloacal route, demonstrating a different pattern of viral shedding dependent of the HPAIV. We found a high variation in the susceptibility to HPAIVs between the different chicken breeds. The birds carrying the genotype AA and AG at position 2032 in chicken Mx gene presented a slightly higher, but not significant, percentage of survival and a statistically significant longer MDT than GG individuals. Our study demonstrated that the severity of HPAI infection is largely dependent of the viral isolate and host factors, underlining the complexity of HPAI infections.

## Highlights

- H7N1 HPAIV is more virulent to chickens than H5N8 HPAIV.
- The lower cloacal excretion of H5N8 suggests a lower adaptation to chickens.
- Huge differences in susceptibility to HPAIVs exist between chicken breeds.

## Introduction

Chickens are highly susceptible to highly pathogenic avian influenza viruses (HPAIVs); however, the severity of infection varies depending on the viral strain. The inoculation of most HPAIVs in chickens causes evident clinical signs (e.g. apathy, nervous signs) and gross lesions (e.g. cutaneous edema, cyanosis of the comb and wattles, haemorrhages in skin), and chickens usually die in a period ranging from 3 to 6 days post-inoculation (dpi). However, some HPAIVs produce a peracute infection that kills birds in a shorter period of time (within 3 dpi) [1, 2]. Differences can be observed even in closely related isolates of the same subtype, indicating that point or few mutations in viral proteins may have a pivotal effect in their virulence [3]. In 2016/2017, the H5N8 HPAIV belonging to clade 2.3.4.4 B of Goose/Guangdong (Gs/GD) H5 lineage was responsible for the fourth intercontinental wave of this lineage. Despite Europe has been affected by the four waves of Gs/GD H5 lineage, the one caused by H5N8 B HPAIV was the largest in magnitude (reported poultry outbreaks and deaths), geographic spread and rapidity of incidents [4, 5]. Since Gs/GD H5N8 B HPAIVs may present altered biological properties in chickens, the pathobiological evaluation of this new viral strain is needed.

The pathogenicity of HPAIVs is also influenced by numerous host factors, including species, age at infection and immune response. Several reports demonstrate that a wide range of susceptibility to HPAIV infection is present between chicken breeds/lines. Some breeds display a comparatively high resistance, whereas other breeds are particularly susceptible [3, 6–10]. Local chicken breeds are generally raised in small-scale farms and in backyards that allow the direct contact with wild and synanthropic birds and their droppings. Consequently, these birds are likely more exposed to AIVs than commercial chicken breeds, which are raised under high biosecurity standards. However, there is a general belief that local chicken breeds are naturally resistant to disease, which is associated with the natural selection over the years by autochthonous pathogens, food availability and harmful climate [11]. This assumption is usually a result of empiric experience at the field, and the results of experimental studies addressing the susceptibility of local chicken breeds to HPAIVs do not always support this theory [10]. To date, there is no information regarding the susceptibility of local Spanish chicken breeds to HPAIVs.

Despite the genetic background that confers higher resistance to HPAIVs in chickens remains unknown, it was reported that the G/A polymorphism at position 2032 in chicken Mx gene (substitution of serine to asparagine at position 631 in the protein) conferred an antiviral effect against AIV infection in vitro [12]. However, several in vitro and in vivo experiments have failed to demonstrate a clear correlation between this polymorphism and inhibition of AIVs or survival after infection, respectively [6, 13–16]. Therefore, the role of Mx in AIVs infections in chickens is still under debate.

Since the pathobiology of HPAIVs in chickens is multifactorial and numerous viral and host factors can largely influence the infection outcome, the aims of this study were to (1) evaluate the pathobiology of a recent H5N8 HPAIV isolated in Spain (Gs/GD lineage, clade 2.3.4.4, Group B) in comparison with a classical H7N1 HPAIV in different local, commercial and experimental chicken lines from Spain with diverse genetic backgrounds; and (2) determine the role of virus factors (differences in the sequence of amino acids in viral proteins between both HPAIVs) and host factors (allele at position 2032 of chicken Mx gene) in the infection outcome.

## Materials and methods

### Viruses

The viruses used in this study were: A/Chicken/Italy/5093/1999 (H7N1), isolated in 1999–2000 during an Italian epidemic that mainly affected Veneto and Lombardia regions (kindly provided by Dr. Ana Moreno from the *Instituto Zooprofilattico Sperimentale della Lombardia e dell’Emilia Romagna*), and A/Goose/Spain/IA17CR02699/2017 (H5N8 clade 2.3.4.4. group B), isolated in Catalonia (Northern Spain) during the 2016/2017 European epizootics. Both viruses are highly pathogenic based on the amino acid sequences at the HA0 cleavage site: PEIPKGSRVRR↓GLF (H7N1) and PLREKRRKR↓GLF (H5N8). Virus stocks were produced in 10 days-old specific-pathogen free (SPF) embryonated eggs. The allantoic fluid was obtained at 24–48 h post-inoculation (hpi), filtered and aliquoted at − 75 °C until use. Serial ten-fold dilutions of the filtered viruses in PBS were used for titration in 10 days-old SPF embryonated eggs. The mean embryo lethal doses (ELD_50_) were determined by Reed and Muench method [17]. The consensus full genome sequences corresponding to the eight segments of the local H5N8 are available in Genbank under accession numbers MK494920 to MK494927 (H5N8).

### Animals and facilities

Fifteen day-old chickens (*Gallus gallus domestica*) of six different local breeds from Spain (*Empordanesa, Penedesenca, Catalana del Prat, Flor d’Ametller, Castellana negra, and Euskal oiloa),* a commercial breed (Ross 308 Broiler) and a commercial-experimental line (SPF White Leghorns) were used. The breeds included in this study were not vaccinated. All local breeds were obtained from local breeders. The local breeds included in this study are common in non-commercial, small-scale flocks, usually in backyards alone or mixed with other species in different regions of Spain. For their characteristics, these breeds are common in chicken contests, and their meat and sub-products are used for self-consumption or sold in local markets because of their added value in the market chain. Specific programs have been established in all included breeds to ensure their conservation [18].

At arrival, the animals were individually identified and placed in negative-pressured high efficiency particulate air (HEPA)-filtered isolators present in Biosecurity Level 3 (BSL-3) facilities of *Centre de Recerca en Sanitat Animal* (*Programa de Sanitat Animal*, IRTA). During the 5 days-acclimation period, serum samples were obtained from all birds to ensure that they were seronegative to AIV by an ELISA competition test (ID-VET, Montpellier, France). Furthermore, OS and CS were collected from 5 randomly selected chickens of each group and confirmed to be negative to AIV RNA by one-step qRT-PCR.

### Experimental design and sampling

After acclimation, 15 chickens of each breed (except for *Castellana negra* and Broilers that consisted in groups of 13 birds, and *Euskal oiola* inoculated with H7N1 that was a group of 10 animals) were intranasally challenged with H7N1 or H5N8 HPAIV diluted in PBS in order to inoculate 10^5^ ELD_50_ in a final volume of 0.05 mL (0.025 mL inoculated in each nostril). Animals belonging to negative control group (2–5 animals/breed) were intranasally inoculated with 0.05 mL of sterile phosphate-buffered saline (PBS).

All birds were monitored daily for clinical signs until 10 dpi. A standardized World Organization for the Animal Health (OIE) clinical scoring system was used [19]. Animals with absence of clinical signs were classified as 0. Birds presenting one of the following clinical signs were considered sick (1) and those showing more than one were considered severely sick (2): respiratory involvement, depression, diarrhea, cyanosis of the exposed skin or wattles, edema of the face and/or head and nervous signs. Birds found dead were scored as 3. For ethical reasons, moribund chickens were anesthetized using the combination of ketamine/xylazine (20 mg/kg body weight, Imalgene 100 and 5 mg/kg body weight, Rompun 20 mg/mL) via the intramuscular route, euthanized by intracardiac injection of pentobarbital overdose (140 mg/kg body weight, Euthasol 400 mg/mL) and scored as dead. The percentage of mortality and mean death time (MDT) were calculated for each virus in all the breeds.

All birds presenting severe clinical signs or found dead were subjected to macroscopic examination. In addition, three chickens of each breed inoculated with H7N1 and H5N8 HPAIVs were killed at 3 dpi using the combination of drugs reported above to collect tissue samples for pathological studies. The selection of birds was biased towards those found dead or presenting evident clinical signs of disease. Two birds of each breed belonging to mock-infected groups were also necropsied at 3 dpi. In order to evaluate viral shedding, oral swabs (OS) and cloacal swabs (CS) were collected from 9 chickens of each breed (selected previously to the inoculation) challenged with H7N1 and H5N8 HPAIVs, and from negative control animals, at 1, 3, 6 and 10 dpi. The same birds were sampled through the experiment.

### Pathological examination and immunohistochemical testing

Tissue samples collected from the chickens necropsied at 3 dpi were immersed in 10% formalin for fixation during 72 h and embedded in paraffin wax. Samples included skin, thymus, pectoral muscle, nasal cavity, trachea, lung, central nervous system, heart, spleen, liver, kidney, proventriculus, gizzard, pancreas, small intestine, large intestine and bursa of Fabricius.

Microtome sections of 3 µm of thickness (Leica RM2255, Nussloch, Germany) from formalin-fixed, paraffin-embedded tissues collected at 3 dpi were processed, stained with haematoxylin and eosin (H/E) and then examined under light microscopy. An immunohistochemical (IHC) technique was performed in the same tissues. Briefly, samples were pretreated with 0.1% protease at 37 °C during 8 min. A mouse-derived monoclonal commercial antibody against the nucleoprotein (NP) of IAVs (ATCC, HB-65, H16L-10-4R5) was used as a primary antibody. The slides were incubated overnight at 4 °C. The samples were then incubated with an anti-mouse secondary antibody conjugated to an HRP-Labelled Polymer (Dako, immunoglobulins As, Denmark). The antigen–antibody reaction was visualized using the chromogen 3,3′-diaminobenzidine tetrahydrochloride. Sections were counterstained with Mayer’s haematoxylin and examined under light microscopy. The positivity in the tissues was semi-quantitatively assessed taking into consideration the percentage of NP-positive and negative cells in the tissue. The samples were classified as follows: no positive cells (−), < 10% positive cells (+), 10–40% positive cells (++), > 40% positive cells (+++) in a tissue section. Positive and negative controls were used. The positive control was a central nervous system from a chicken experimentally infected with H7N1 HPAIV [20], and the negative control consisted in the same tissue incubated with PBS instead of the primary antibody and also the tissues collected from negative control chickens.

### Viral RNA quantitation in swabs

Swabs were placed in 0.5 mL of sterile PBS enriched with Penicillin–Streptomycin (Thermo Fisher Scientific, Waltham, Massachusetts, USA) and Nystatin (Sigma-Aldrich, Missouri, USA) at a final concentration of 6%. Swabs were conserved at − 75 °C until further use. Viral RNA was extracted using Nucleospin RNA virus kit (Macherey–Nagel, Düren, Germany), following manufacturer’s instructions. A highly conserved region of 99 bp present in IAV M1 gene was amplified and detected by one-step Taqman RT-PCR technique in Fast7500 equipment (Applied Biosystems, Foster City, CA, USA), using the same primers and probe as well as conditions of amplification previously described [21, 22]. To extrapolate the genome equivalent copies (GEC) present in the swabs, a standard curve obtained by amplification of the same region of M1 gene was used. Briefly, the amplified region was ligated in pGEM-T vector (Promega, Madison, Wisconsin, USA). The ligation product was purified using MinElute Reaction Cleanup Kit (Qiagen, Valencia, CA, USA) and transfected into electrocompetent *E.coli* cells (Thermo Fisher Scientific, Waltham, Massachusetts, USA) by electroporation. The recombinant plasmid was purified from transformed colonies using NucleoSpin Plasmid (Macherey–Nagel, Düren, Germany) and quantified in Biodrop (Biodrop µLite, Cambridge, England). GEC were calculated using DNA Copy Number Calculation (Thermo Fisher Scientific, Waltham, Massachusetts, USA). Serial ten-fold dilutions were used to obtain the standard curve. The limit of detection of the technique was 1.89Log_10_ GEC in both OS and CS.

### RFLP-PCR Mx

Prior to infection, total blood in a 1:1 ratio with anticoagulant (Alsever’s solution, Sigma-Aldrich, Missouri, USA) was obtained from all chickens belonging to H7N1 and H5N8 HPAIV-inoculated groups. Genomic DNA was isolated from 10 µL anticoagulated blood using a standard DNA purification kit (DNeasy Mini Kit, Qiagen, Valencia, CA, USA), following manufacturer’s instructions. To avoid RNA contamination, samples were treated with RNase (RNase A, Qiagen, CA, USA). As described by Sironi et al. [23], the following primers were used to amplify a 299 pb region in exon 14 of chicken Mx gene: forward 5′-GCACTGTCACCTCTTAATAGA-3′ and reverse 5′-GTATTGGTAGGCTTTGTTGA-3′. PCR reaction mixture included 60 ng genomic DNA, 10 µmol of each primer, 10 × buffer, 1.5 mM MgCl_2_, 0.2 mM of each dNTP and native Taq DNA polymerase (5 U/µL) (Taq DNA Polymerase, native, ThermoFisher Scientific, Massachusetts, USA) in a final volume of 25 µL. Mx region was amplified in GeneAmp PCR System 9700 equipment (Applied Byosistems, CA, USA) as follows: 95 °C for 10 min, 35 cycles of 94 °C for 1 min, annealing at 53 °C for 1 min, and 72 °C for 1 min, and a final extension step at 72 °C for 10 min.

Five μL of PCR products were run in a 2% agarose gel in 1X TAE buffer with ethidium bromide to confirm the presence of a specific band at 299 pb. PCR products were incubated at 37 °C during 16 h with a restriction enzyme (Hpy8I-MjaIV, 10 U/µL, Thermo Fisher Scientific, Massachusetts, USA), following manufacturer’s instructions. The restriction enzyme (5′-GTN|NAC-3′) cleaves the sequence 2 pb downstream of the Mx polymorphism in presence of guanine (G), whereas the product is not cut in case of an adenine (A) at this position. Digestion products were visualized in a 2% agarose gel in 1X TAE buffer with EtBr. Animals were classified in homozygous-resistant genotype (AA), heterozygous-intermediate genotype (AG) and homozygous-susceptible genotype (GG).

The proportion of birds dead at the end of the study by genotype groups were compared using the Pearson’s Chi square test. Then, post hoc pairwise comparisons with Bonferroni corrections were carried out [24]. Also, for the animals that succumbed to infection, the MDT by genotypes were compared. First, the normality of the data was assessed using the Shapiro–Wilk test. Then groups were compared using either the Anova test (in case of normally distributed data), or the Kruskal–Wallis test (in case of non-normally distributed data). Finally, post hoc comparisons were carried out using the Tukey test (for normally distributed data), or Dunn’s test with Bonferroni correction (in case of non-normally distributed data). All calculations were carried out using R statistical software (http://cran.r-project.org/).

## Results

### Clinical signs and mortality

**Figure 1:**
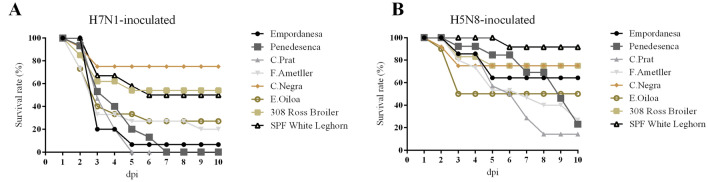
Survival curves of the different chicken breeds experimentally inoculated with H7N1 (**A**) or H5N8 (**B**) HPAIVs at a dose of 10^5^ ELD_50_.

The mortality and percentage of clinical signs after experimental infection with either H7N1 or H5N8 HPAIVs in the different chicken breeds are presented in Figure 1 and Table 1. Severe clinical signs were observed in H7N1 and H5N8 HPAIVs-inoculated chickens in all breeds, but the frequency varied depending on the viral isolate and the chicken breed. At 2 dpi, several chickens of different breeds inoculated with H7N1 HPAIV presented severe apathy, were prostrated or found dead without previous evident clinical signs. Few chickens inoculated with H5N8 HPAIV also presented severe apathy at 2 dpi and were consequently euthanized. From 2 dpi and lasting until 9 (H7N1) or 10 dpi (H5N8), severe clinical signs were detected in several chickens at different times post-inoculation. The main clinical signs observed in both HPAIV infections were moderate apathy that progressed to prostration, and less frequently subcutaneous oedema, cyanosis of the comb and wattles and nervous signs (ataxia, circling, tremor and head shaking). The percentage of animals presenting prostration and nervous signs was higher in chickens challenged with H7N1 HPAIV than in those inoculated with H5N8 HPAIV (55% and 8% for H7N1 versus 35% and 4% for H5N8). Similarly, the onset of nervous signs was earlier in H7N1 HPAIV-inoculated groups than in the inoculated with H5N8 HPAIV (3 dpi versus 5 dpi). Moreover, H7N1 HPAIV produced a higher mortality rate in chickens than H5N8 HPAIV (70 versus 47%, respectively), as well as a shorter MDT (3,3 versus 4,9 dpi, respectively).

**Table 1:** Clinical signs, mortality and MDT of the different chicken breeds challenged with H7N1 or H5N8 HPAIVs.

Virus	Parameter	*Empordanesa*	*Penedesenca*	*C. Prat*	*F. Ametller*	*C. Negra*	*E. Oiloa*	308 Ross Broiler	SPF White Leghorn	Mean
H7N1	Percentage of dead birds	93	100	100	80	25	73	46	40	70
	Mean death time (dpi)	3.3	4.2	3.3	3.3	2.7	3	3	3.8	3.3
	Clinical signs (%)									
	Severe apathy	93	73	67	40	15	67	31	40	55
	Cutaneous edema/cyanosis	7	20	7	0	0	0	0	7	5
	Nervous signs	0	7	20	0	8	20	8	0	8
H5N8	Percentage of dead birds	33	76	85	73	25	50	25	8	47
	Mean death time (dpi)	4.2	7.9	5.8	5.7	2.7	2.8	3.7	6	4.9
	Clinical signs (%)									
	Severe apathy	7	40	40	53	23	40	8	67	35
	Cutaneous edema/cyanosis	0	7	7	7	0	0	0	8	4
	Nervous signs	0	7	0	0	0	0	0	0	4

Regarding breeds, *Castellana negra*, Broiler and SPF chicken breeds presented a lower frequency of clinical signs and considerably lower mortality rates (≤ 50%) than *Penedesenca, Catalana del Prat, Flor d’Ametller and Euskal Oilo*a (≥ 50%) breeds in both H7N1 and H5N8 HPAIVs-inoculations. Only *Empordanesa* breed presented differing susceptibility depending on the virus tested (93 and 33% mortality after challenge with H7N1 and H5N8, respectively). *Catalana del Prat and Penedesenca* presented the highest incidence of nervous signs and cutaneous edema among all the tested breeds, respectively (Table 1).

### Gross lesions

Macroscopic examination of the chickens inoculated with H7N1 or H5N8 HPAIVs revealed similar lesions with both viruses in all breeds. At 2 dpi, few chickens inoculated with H7N1 HPAIV exhibited multifocal haemorrhages in proventriculus and gizzard, whereas non-evident lesions were present in the chickens inoculated with H5N8 HPAIV. From 3 dpi to the end of the study, the most common findings in the chickens inoculated with H7N1 and H5N8 HPAIVs were multifocal petechiae and necrotic areas in pancreas, and/or multifocal petechiae in proventriculus, gizzard and in the proventriculus-gizzard junction. Congestion in central nervous system was also a common finding. Less frequently, several chickens exhibited hemorrhages of variable intensity in skin (e.g. legs), subcutaneous edema, lung consolidation and diffuse congestion in internal organs. At 10 dpi, one chicken inoculated with H5N8 HPAIV presented multifocal petechias in bursa of Fabricius. No macroscopic lesions were observed in negative control birds.

### Histopathological findings

Microscopic examination of the tissues collected from dead or severely-affected chickens at 3 dpi revealed evident lesions of variable intensity in all breeds in mostly all the collected organs. However, we detected differences in the severity and viral replication in the different tissues between H7N1 and H5N8 HPAIV-inoculated chickens (Additional file 1, Additional file 2, respectively). In both viral infections, the predominant microscopic lesions in the tissues were areas of necrosis and haemorrhages with mixed inflammatory infiltrate (macrophages, lymphoplasmacytic cells and heterophils). The extension and severity of microscopic lesions correlated well with the intensity of IAV antigen (IHC techniques) in the tissues. The main organs affected were similar in all the chicken breeds inoculated with the same virus.

**Figure 2:**
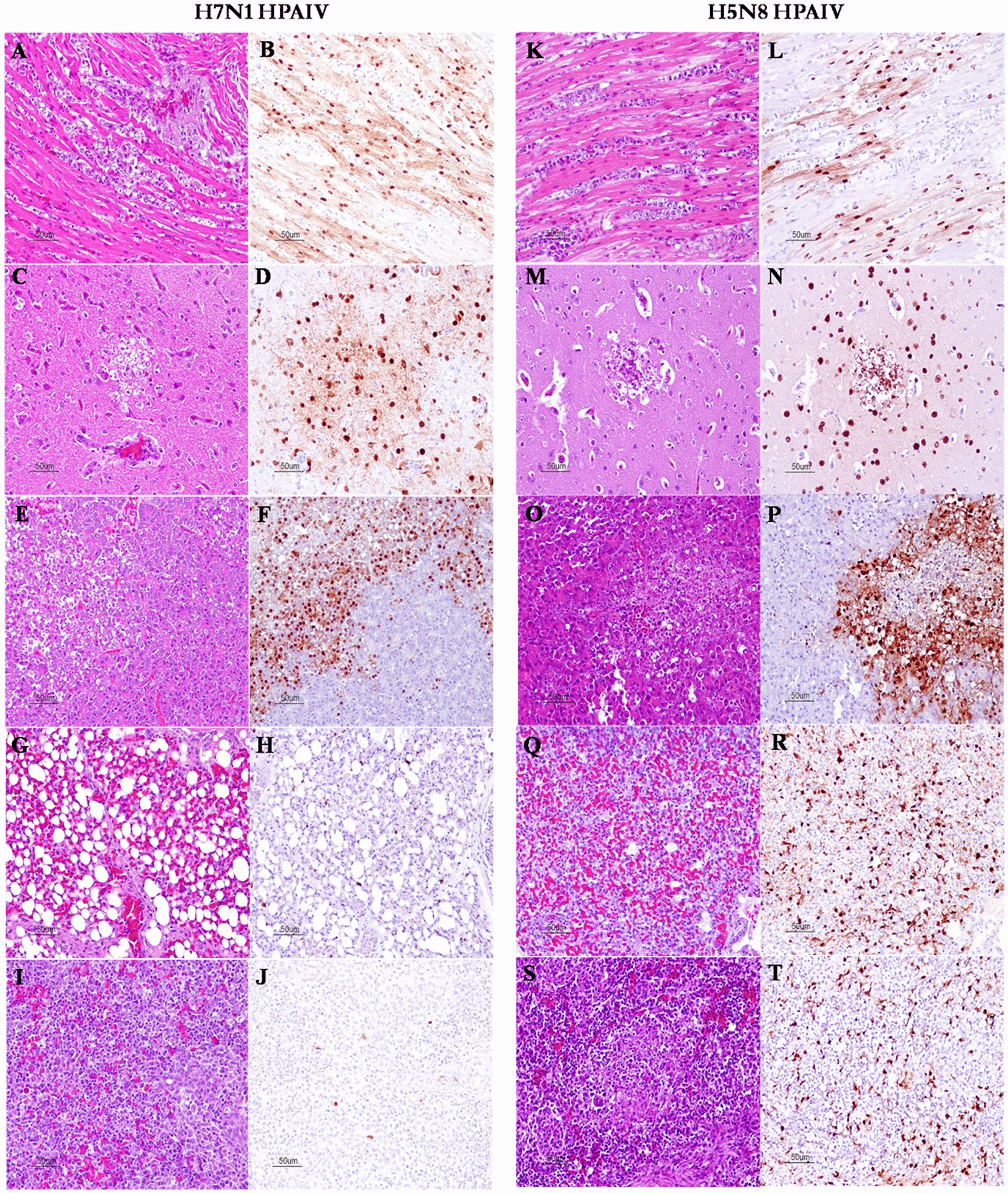
Microscopic lesions (HE staining) and viral replication (IHC staining) at 3 dpi in several organs obtained from chicken breeds experimentally inoculated with H7N1 and H5N8 HPAIVs. Myocardium **A**, **B** (H7N1 infected), **K**, **L** (H5N8 infected): multifocal necrosis of myocardiocytes with inflammatory infiltrate (**A**, **K**) and NP-positive myocardiocytes and inflammatory cells (**B**, **L**). CNS **C**, **D** (H7N1 infected) **M, N** (H5N8 infected): multifocal areas of necrosis in cerebral hemispheres (**C**, **M**), widespread NP-positive neurons and glial cells (**D**, **N**). Pancreas **E**, **F** (H7N1 infected), **O**, **P** (H5N8 infected): diffuse area of necrosis in pancreatic acinar cells (**E**, **O**) associated with widespread NP-positive cells in necrotic areas and surrounding acinar pancreatic cells (**F**, **P**). Lung **G**, **H** (H7N1 infected), **Q**, **R** (H5N8 infected): mild increase of cellularity (mixed inflammatory cells) in air capillaries interstitium (**G**) and few NP-positive inflammatory cells (**H**). Severe increase of increase of cellularity in air capillaries interstitium, focal areas of necrosis in pneumocytes, microthrombi and diffuse oedema (**Q**), widespread NP-positive cells in inflammatory cells, endothelial cells and air capillary cells (**R**). Spleen **I**, **J** (H7N1 infected), **S**, **T** (H5N8 infected): non-apparent lesions (**I**) and few NP-positive lymphoid-cells (**J**). Areas of necrosis with mixed inflammatory cell infiltration (**S**), widespread NP-cells in inflammatory and endothelial cells (**T**).

In H7N1 HPAIV-inoculated chickens, the most relevant microscopic lesions and viral replication were observed in heart, followed by central nervous system and pancreas. Viral replication in the heart was associated with fiber degeneration/necrosis and hyalinization of myocardiocytes mixed with mild inflammatory cell infiltration (mainly macrophages) (Figures 2A, B). In the central nervous system, non-suppurative encephalitis consisting in multifocal areas of necrosis in cerebral hemispheres, intense spongiosis, neuronal chromatolysis and gliosis were commonly observed (Figures 2C, D). In cerebellum, chromatolysis of Purkinje neurons was a common finding. The lesions observed in the pancreas were multifocal areas of lytic necrosis of exocrine gland cells (Figure 2E, F). The remaining tissues generally presented mild necrotic and/or inflammatory lesions and few positive cells, such as lung and spleen (Figures 2G–J, respectively).

The chickens inoculated with H5N8 HPAIV presented the most severe lesions and intense viral replication in the lung and heart, but evident lesions and high viral replication were also detected in spleen, thymus, central nervous system, nasal cavity, gizzard, pancreas and liver. The lesions in the heart, central nervous system and pancreas were similar to those described in H7N1 HPAIV-inoculated chickens (Figures 2K–P, respectively). In the lung, interstitial pneumonia consisting on moderate to severe increase of the cellularity (macrophages and lymphoid cells) in air capillaries and focal areas of necrosis associated with intense viral replication was commonly observed (Figures 2Q, R). In lymphoid tissues, including spleen, thymus and bursa of Fabricius, multifocal areas of necrosis/apoptosis of variable intensity in mononuclear cells were present. Particularly, diffuse necrotic areas and widespread viral replication were present in the spleen of one chicken (Figures 2S, T). Several animals also presented multifocal areas of necrosis in respiratory and olfactory epithelial cells in the nasal cavity. Multifocal areas of necrosis in glandular cells with mixed inflammatory cell infiltration, muscular cell degeneration and necrosis of lymphoid tissue were detected in gizzard and proventriculus. In the liver, we detected focal areas of necrosis with mild distention of hepatic sinusoids. The remaining tissues (skin, pectoral muscle, kidney and intestines) presented mild multifocal necrotic and/or inflammatory lesions and few positive cells.

### Viral shedding

**Figure 3:**
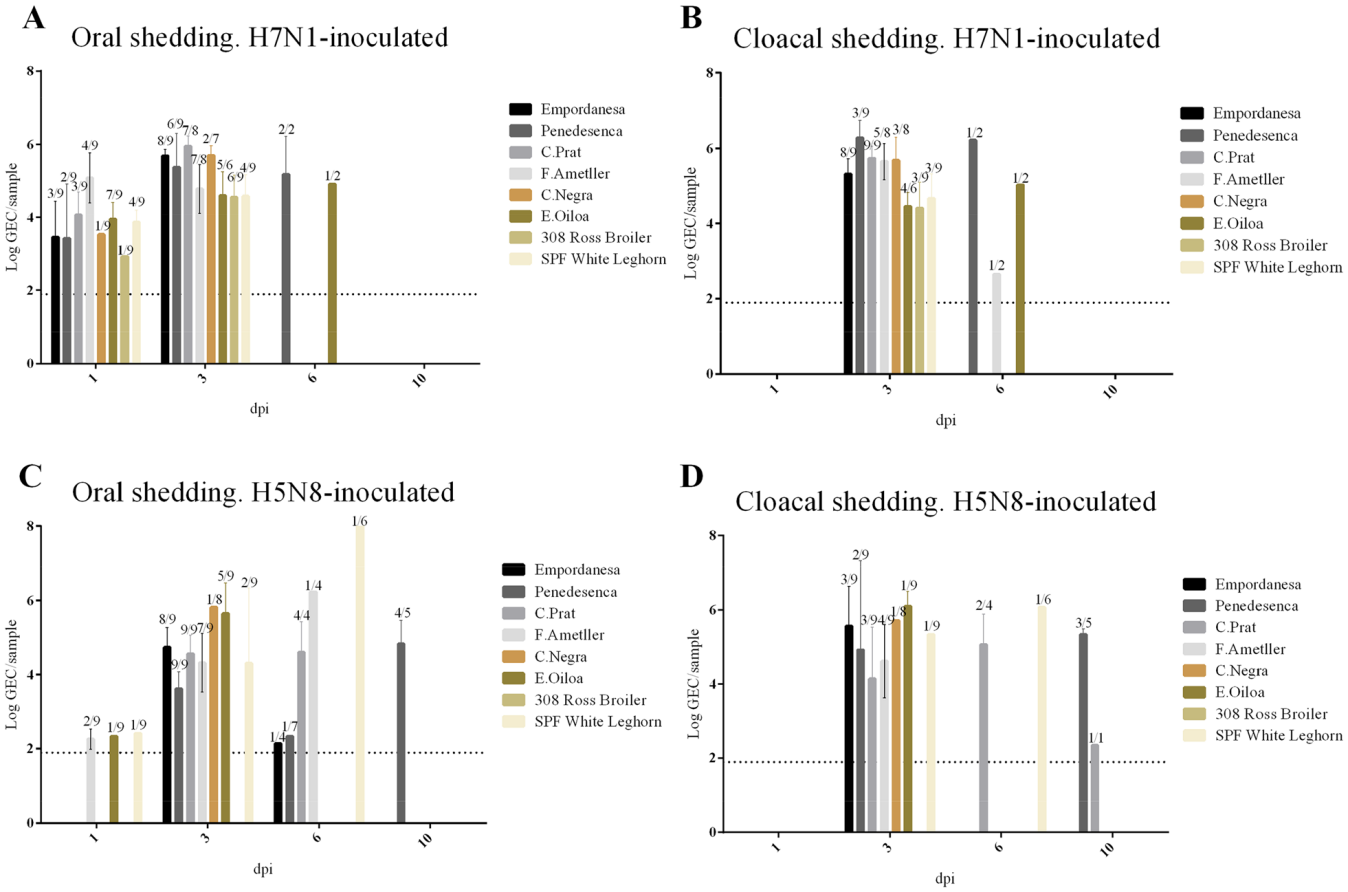
Viral titers expressed as Log_10_ GEC in OS and CS obtained from chickens inoculated with H7N1 (**A**, **B**) or H5N8 (**C**, **D**) HPAIVs at different time points. Viral titers are represented as the mean values ± SEM. The numbers above the columns represent the number of chickens shedding virus out of the total sampled. GEC: Genome equivalent copies; Dpi: day post-inoculation.

Differences in the viral shedding between H7N1 and H5N8 HPAIV-inoculated chickens were detected (Figures 3A–D, respectively). High viral excretion by both oropharyngeal and cloacal routes was detected in chickens inoculated with H7N1 HPAIV. At 1 dpi, viral RNA was detected in several OS but not in CS. The peak of shedding occurred at 3 dpi. By 6 dpi, few birds presented detectable levels of virus RNA in OS and CS, but the levels detected were similar with those collected at 3 dpi. No viral RNA was present in the OS and CS collected at 10 dpi. Regarding H5N8 HPAIV-inoculated groups, a low number of OS presented detectable levels of viral RNA at 1 dpi. The proportion of positive OS and levels of viral RNA peaked at 3 dpi. In contrast, a low number of birds inoculated with H5N8 HPAIV presented cloacal shedding at the different dpi tested. However, the levels of viral RNA in the positive CS at 3 dpi were similar to those present in OS. By 6 and 10 dpi, viral shedding was still detected in several birds by both the oral and cloacal routes and in some samples, the levels were high.

Both H7N1 and H5N8 HPAIV-inoculated chickens presented similar oral shedding, as demonstrated by a similar number of chickens shedding virus (70% and 58% by 3 dpi, respectively) and mean levels of viral RNA in OS (5.1 and 4.6 Log_10_ GEC by 3 dpi, respectively). The mean levels of viral RNA in CS were also similar between H7N1 and H5N8-HPAIV inoculated groups (5.3 and 5.2 Log_10_ GEC by 3 dpi, respectively). However, a higher number of chickens inoculated with H7N1 HPAIV presented cloacal excretion in comparison with those inoculated with H5N8 HPAIV (56% and 21% by 3 dpi, respectively).

Despite the levels of viral RNA in OS and CS obtained through the experiment were quantitatively similar in all the chicken breeds included in the study, a higher proportion of chickens of the *Empordanesa, Penedesenca, Catalana del Prat, Flor d’Ametller and Euskal Oiloa* breeds excreted virus by oral and cloacal routes in both viral infections than those belonging to *Castellana negra*, Broiler and SPF breeds.

### Polymorphism at position 2032 of Mx gene and association with infection outcome

We found marked differences regarding genotype and allele distribution at position 2032 of chicken Mx gene in the breeds included in this study (Table 2). The three genotypes AA, AG, GG were present in *Empordanesa, Penedesenca, Catalana del Prat and Castellana negra* breeds with variable frequency. The heterozygous genotype (AG) was the predominant in *Empordanesa and Catalana del Prat* breeds. *Penedesenca* presented a higher frequency of the homozygous-resistant genotype (AA), whereas *Castellana negra* had more frequently the homozygous-susceptible genotype (GG). Almost all *Flor d’Ametller* and *Euskal Oiloa* chickens presented the homozygous-susceptible genotype (0.939 and 0.923, respectively), and any the resistant genotype. SPF chickens presented predominantly the homozygous-resistant genotype (AA), and in a minor amount the heterozygous one, but not the homozygous-susceptible one. Broiler chickens were fixed for the homozygous-susceptible genotype (GG). Overall, the average genotype frequency of the susceptible genotype GG (0.50) was higher than AG (0.29) and AA (0.21) genotypes (data not shown). The A allele was present in all the chicken breeds except in Broilers, but the frequency varied from 0.030 and 0.038 in *Flor d’Ametller* and *Euskal oiloa* breeds, respectively, to 0.903 in SPF chickens. G allele was present in all the chicken breeds. In this case, the range of frequencies varied in the interval of 1.00 present in Broiler chickens to 0.097 in SPF chickens. G allele (0.65) was more predominant in the chicken population tested than A allele (0.36) (data not shown).

**Table 2:** Genotype AA, AG and GG and allele frequencies of A and G alleles in exon 14, position 2032 of chicken Mx gene in different chicken breeds.

Breed	Genotype frequency			Allele frequency	
	AA	AG	GG	A	G
*Empordanesa*	0.129	0.452	0.419	0.355	0.645
*Penedesenca*	0.485	0.364	0.152	0.667	0.333
*Catalana del Prat*	0.156	0.750	0.094	0.531	0.469
*Flor d’Ametller*	0.000	0.061	0.939	0.030	0.970
*Castellana negra*	0.100	0.400	0.500	0.300	0.700
*Euskal oiloa*	0.000	0.077	0.923	0.038	0.962
308 Ross Broiler	0.000	0.000	1.000	0.000	1.000
SPF White Leghorn	0.806	0.194	0.000	0.903	0.097

*AA* resistant genotype, *AG* heterozygous genotype, *GG* susceptible genotype

In order to study the association of Mx genotypes at position 2032 with percentage of survival at the end of the study and mean days of death in the birds that succumbed to infection, statistical analyses were performed. The differences in the proportion of dead birds (containing both HPAIVs) were not statistically significant among genotypes. However, the Kruskal–Wallis test indicated statistically significant differences in the MDTs among genotype groups (p value < 0.0001). Post-hoc pairwise using the Dunn’s test with Bonferroni correction indicates differences were statistically significant between groups AA and GG (5 versus 3.6 dpi, respectively; p = 0.0015), and between groups AG and GG (4.7 versus 3.6 dpi, respectively; p = 0.0006).

## Discussion

Available data demonstrates that the susceptibility to HPAIVs varies largely depending of the viral isolate and the genetic background of the host. In order to evaluate the existence of viral-and host-dependent differences in HPAIV infections in chickens, we selected a classical HPAIV (H7N1 isolated from a chicken in Italy) and a recent HPAIV of the Gs/GD H5 lineage (H5N8 Gs/GD clade 2.3.4.4 group B isolated from a domestic goose in Spain) and assessed their pathobiology in a broad spectrum of chicken breeds from Spain (local and commercial breeds).

Both HPAIVs used in this study were highly virulent to chickens, as expected based on the presence of a multi-basic cleavage site in the HA protein and demonstrated experimentally by the severe clinical signs and fatal outcomes observed through the experiment. However, H7N1 and H5N8 HPAIVs differed in the progression of the disease they caused in chickens. With the highest frequency of prostration and neurological signs, highest mortality rates and shortest MDT, H7N1 HPAIV is more virulent to chickens than H5N8 HPAIV. The viral shedding pattern also varied between H7N1 and H5N8 HPAIVs. The differences in oral excretion between H7N1 and H5N8-inoculated chickens were minor. However, a low number of chickens inoculated with H5N8 HPAIV presented cloacal excretion. Despite some birds were still shedding at the end of the study, our findings suggest the potential for decreased horizontal transmission efficiency of Gs/GD H5N8 clade 2.3.4.4 B HPAIV among chickens. In concordance with our results, previous studies demonstrate that Italian H7N1 HPAIV exhibit high virulence and transmissibility in several galliformes species [20, 25, 26], whereas several Gs/GD clade 2.3.4.4 H5Nx reassortants (including H5N8 HPAIVs) cause lower mortalities, longer MDTs and present lower transmissibility in chickens compared to their ancestral Gs/GD H5N1 HPAIVs [8, 27–29]. These results confirm the variable pathogenicity and potential transmissibility of HPAIVs of different lineages and host-origin in the chicken species.

The lower frequency of clinical signs and mortality, longer MDT and reduced excretion in H5N8 HPAIV-inoculated chickens in comparison with those inoculated with H7N1 HPAIV suggest a lower affinity and/or adaptation of H5N8 HPAIV to chickens. This could have partially contributed to the limited number of H5N8 HPAIV outbreaks in chicken holdings during the 2016–2017 epidemics in Europe (12% of the total reported outbreaks), in comparison with its detections in waterfowl holdings, including in Spain [5]. However, different genotypes of H5N8 HPAIV circulated in Europe at that time [30]. Therefore, important differences in the biological properties and virulence of H5N8 HPAIV between European strains may exist. The production characteristics of this species could also be an important factor of the comparatively lower incidence of H5N8 outbreaks. Migratory wild birds are thought to have played a pivotal role in the worldwide dissemination of Gs/GD H5N8 HPAIVs [31]. Since chicken production in Europe in mostly intensive farms presents high biosecurity standards, the low detection in chicken holdings during the 2016/2017 epidemics may also be due to a low exposure to the virus.

Despite the different course of infection caused by H7N1 and H5N8 HPAIVs, the clinical signs and macroscopic lesions in the severely affected chickens were similar and consistent with HPAIV infection. The viral antigen detected in mostly all collected organs in H7N1 and H5N8 HPAIVs-inoculated chickens that succumbed to infection demonstrates the widespread dissemination of both HPAIVs. However, the intensity of replication and associated microscopic lesions in the different tissues were virus-dependent, indicating differences in their tissue tropism. Chickens inoculated with H7N1 presented severe lesions and high viral replication in heart, central nervous system and pancreas. In addition to those observed in H7N1-inoculated birds, inflammatory and necrotizing lesions associated to intense viral replication were detected in the lungs and primary lymphoid organs collected from chickens inoculated with H5N8 HPAIV. Widespread staining was generally detected in lymphocytes in these tissues, indicating a high avidity of H5N8 HPAIV for lymphoid cell populations. The cause of death in the chickens that succumbed to infection after H7N1 and H5N8 HPAIV inoculation appears to be the result of the multi-organ replication of these viruses. However, H5N8 HPAIV presents a reduced neurotropism, a hallmark of HPAIV pathogenesis, based on the comparatively lower amounts of viral antigen and lesions detected in the brain of H5N8 HPAIV-inoculated chickens in comparison with those inoculated with H7N1 HPAIV. The reduced neurotropism could be a reason of the lower mortalities caused by H5N8 HPAIV. Other mechanisms of pathogenicity not evaluated in the present study may also impact the differences in virulence between H7N1 and H5N8 HPAIVs in chickens, including an aberrant innate immune response in H7N1 HPAIV-inoculated chickens after infection [32].

Particular amino acids in specific positions of PB2, PB1, PA, NP, MP, NS1 and NEP/NS2 proteins sequence have been associated with increased pathogenicity and transmissibility of HPAIVs in chickens [33–35]. Even closely related HPAIV isolates differ in their virulence in chickens, indicating that few mutations in the viral genome may produce significant biological effects [3]. Although there is no evidence of sustained circulation of Spanish H5N8 HPAIV in galliformes species, the Italian H7N1 HPAIV emerged from a LPAIV precursor that had been circulating in gallinaceous poultry for several months [36]. Therefore, H7N1 HPAIV may present markers of adaptation and/or virulence in internal and non-structural proteins that are lacking in H5N8 HPAIV. With the exception of NS protein, the amino acid identity in internal and non-structural proteins was high (˃98%). Several amino acid substitutions associated with increased virulence of HPAIVs in chickens or chicken-derived cells were present in PB2 (123E), PB1 (3 V, 38Y), PA (672L), NP (105 V, 184 K), M1 (43 M) and NS1 (106 M, 125D) proteins in both H7N1 and H5N8 HPAIVs [34, 35, 37–43]. Only two differing amino acids in the sequence of the proteins between H7N1 and H5N8 HPAIVs have been reported to have a biological effect in the chicken species: 103F and 114S that are present in NS1 protein of H5N8 HPAIV. These mutations are associated with inhibition of host gene expression [42]. However, they were reported in a LPAIV strain and their effect in vivo was not evaluated. Therefore, the differences in infection outcome between the two HPAIVs may be due to a single or a combination of amino acid substitutions whose effects have not been yet characterized. Since they belong to different subtypes, the HA and NA surface glycoproteins are also expected to have played a critical role in the differential outcomes. Actually, we detected that H7N1 HPAIV presented an amino acid substitution in HA (388T, H5 numbering) which is associated with increased pathogenicity in chickens [37]. However, the biological implications of the amino acid substitutions reported here require further evaluation. H7N1 HPAIV presents a stalk deletion in the NA protein, commonly observed after a transmission of HPAIV from waterfowls to poultry and a known major virulence determinant of HPAIVs in chickens [44].

The outcome after infection with HPAIVs is also largely influenced by host factors. Several reports demonstrate a wide range of susceptibilities to AIVs among breeds and lines of chickens. Specifically, significant variations in mortalities after experimental inoculation with Gs/GD H5N1, H5N6, H5N8 HPAIVs [3, 7–10, 45, 46] and the Italian HPAIV H7N1 [6] have been reported. We then evaluated the existence of breed-related differences in the susceptibility to HPAIVs in a broad range of local chicken breeds, and in two commercial breeds. In the present study, four breeds (*Empordanesa, Penedesenca, Catalana del Prat, Flor d’Ametller*, *Euskal Oiloa*) were highly susceptible to HPAIV infection, whereas three breeds (*Castellana negra*, Broiler and SPF chickens) were considerably more resistant. The breeds that were more resistant exhibited less frequency of severe clinical signs, lower mortality rates and lower number of animals shedding virus to both HPAIVs infections than susceptible breeds, demonstrating that the genetic background of particular chicken breeds confer a higher natural resistance to diverse HPAIVs subtypes. Moreover, two of the highly susceptible breeds presented a higher incidence of cutaneous edema and nervous signs, suggesting that the clinical presentation may vary to some extent dependent of the breed.

Local chicken breeds are believed to be more resistant to disease as a result of the natural selection by autochthonous pathogens and minor artificial selection towards productive-associated genes [11]. However, almost all the local chicken breeds included in our study were highly susceptible to both HPAIVs. This is in concordance with previous reports [10], demonstrating that local breeds do not necessarily present an improved resistance to infectious diseases. Since these breeds are usually raised in backyards in the absence or few biosecurity measures and Spain is located within natural migratory routes between Eurasia and Africa, these particular breeds are expected to be highly vulnerable to infection with HPAIVs carried by migratory birds. Because of that, local chicken breeds could act as sentinels for HPAIV environmental contamination. Despite the susceptibility of local breeds to HPAIVs, their role in the global epidemiology of HPAIVs is much less evident. For instance, the role of backyard poultry on H5N8 epidemiology was suggested to be limited [47].

The genetics of resistance to HPAIVs remains unknown. However, particular alleles present in immune response-related genes have shown a positive correlation with antiviral activity [48, 49]. Mx proteins are induced by type 1 interferons and interfere with viral functions by inhibiting viral polymerases [50]. The substitution of serine with asparagine at position 631 of Mx protein, which is produced by a particular non-synonymous G/A polymorphism in exon 14 of chicken Mx gene, was associated with higher antiviral activity in vitro [48]. In vitro and in vivo studies do not always show a clear correlation between this allele and inhibition of AIV replication and/or survival after HPAIV infection, respectively [6, 13–16]. Therefore, the impact of this particular amino acid substitution is still unclear. In the present study, we evaluated the genotype frequencies of that particular polymorphism in the different chicken breeds and evaluated their association with infection outcome. Similar to in other studies reporting high diversity in genotype and allele frequency in that position between breeds [51], we detected huge differences in the frequency of the three genotypes among the spectrum of local, commercial and experimental breeds included in our study. As reported previously, resistant and susceptible genotypes appear to be the predominant in White Leghorns and Broilers, respectively [52, 53]. In contrast, the results in local breeds were more variable, which could be associated with the higher genetic diversity generally present in unselected breeds. Overall, the susceptible G allele prevails in the Spanish chickens tested, while the resistant A allele was the predominant in two Indian native chicken breeds [54], a native chicken breed from Indonesia [55] and 2 chicken breeds and 7 strains from Egypt [56]. The statistical analyses showed that the different genotypes in the target Mx region were not associated with significant differences in mortality ratios. However, the birds carrying the AA and AG presented a statistically significant longer MDT than those carrying the GG genotype. In concordance with our results, the study carried out by Ewald et al. [57] observed that chickens homozygous for AA allele presented a delayed MDT. These suggest that the presence of an asparagine at position 631 in Mx protein may result in a higher antiviral effect response of Mx protein against HPAIVs, but, as shown in our studies, the biological implications of this change in vivo are probably limited.

This study represents an exhaustive characterization of the pathobiology of two HPAIVs in a broad range of chicken breeds. Our results demonstrate that the outcome after infection with HPAIVs is influenced by numerous tightly interconnected factors, including the viral isolate, the genetic background of the breed and particular alleles in genes encoding antiviral proteins, underlining the complexity of HPAIV infections. A proper surveillance, education of caretakers and biosecurity measures in commercial but also in local chicken holdings are required to early detect the circulation of HPAIVs in the territory.

## Supplementary information

**Additional file 1.** Average distribution of NP-positive cells and associated lesions in tissues collected at 3 dpi from different chicken breeds inoculated with H7N1 HPAIV. n=3/group. -: no positive cells, +: <10% positive cells, ++: 10-40% positive cells, +++: >40% positive cells. E: *Empordanesa,* P: *Penedesenca*, C: *Catalana del Prat*, F: *Flor d’Ametller*, N: *Castellana Negra*, O: *Euskal Oiloa*, B: 308 Ross Broiler, S: SPF White Leghorn.

**Additional file 2.** Average distribution of NP-positive cells and associated lesions in tissues collected at 3 dpi from different chicken breeds inoculated with H5N8 HPAIV. n=3/group. -: no positive cells, +: <10% positive cells, ++: 10-40% positive cells, +++: >40% positive cells. E: *Empordanesa,* P: *Penedesenca*, C: *Catalana del Prat*, F: *Flor d’Ametller*, N: *Castellana Negra*, O: *Euskal Oiloa*, B: 308 Ross Broiler, S: SPF White Leghorn.

## Data Availability

Not applicable.

## Abbreviations

AIVs: avian influenza viruses
BSL-3: biosecurity level 3 containment
c-ELISA: competitive ELISA
CS: cloacal swab
DAB: 3,3′-diaminobenzidine tetrahydrochloride
Dpi: day post-infection
ELD_50_: median embryo lethal dose
FFPE: formalin-fixed, paraffin-embedded
Gs/GD: Asian-origin goose/Guangdong H5 lineage
HA: hemagglutinin
H/E: hematoxilin-eosin
HEPA: high efficiency particulate air
HPAI: highly pathogenic avian influenza
Hpi: hours post-inoculation
HPAIVs: highly pathogenic avian influenza viruses
IHC: immunohistochemistry
M: matrix protein
MDT: mean death time
NA: neuraminidase
NP: nucleoprotein
NS: non-structural protein
OIE: World Organization for the Animal Health
OS: oral swab
PBS: phosphate buffered saline
PB1: polymerase basic protein 1
PB2: polymerase basic protein 1
PA: polymerase acidic protein
qRT-PCR: quantitative reverse-transcription PCR
SPF: specific-pathogen free
