# Supplementary material for: Pathobiology of the highly pathogenic avian influenza viruses H7N1 and H5N8 in different chicken breeds and role of Mx 2032 G/A polymorphism in infection outcome
Source: Vet Res. 2020 Sep 10;51:113. doi: 10.1186/s13567-020-00835-4 (PMC7488313; doi:10.1186/s13567-020-00835-4)
Supplement: Supplementary file 2 — Additional file 2. Average distribution of NP-positive cells and associated lesions in tissues collected at 3 dpi from different chicken breeds inoculated with H5N8 HPAIV. n=3/group. -: no positive cells, +: <10% positive cells, ++: 10-40% positive cells, +++: >40% positive cells. E: Empordanesa, P: Penedesenca, C: Catalana del Prat, F: Flor d’Ametller, N: Castellana Negra, O: Euskal Oiloa, B: 308 Ross Broiler, S: SPF White Leghorn. [file 13567_2020_835_MOESM2_ESM.docx]

**Additional file 2.** Average distribution of NP-positive cells and associated lesions in tissues collected at 3 dpi from different chicken breeds inoculated with H5N8 HPAIV**.** n=3/group. -: no positive cells, +: <10% positive cells, ++: 10-40% positive cells, +++: >40% positive cells. E: *Empordanesa,* P: *Penedesenca*, C: *Catalana del Prat*, F: *Flor d’Ametller*, N: *Castellana Negra*, O: *Euskal Oiloa*, B: 308 Ross Broiler, S: SPF White Leghorn.

| **H5N8** | **E** | **P** | **C** | **F** | **N** | **O** | **B** | **S** | **NP+ cell types** | **Microscopic lesions** |
| --- | --- | --- | --- | --- | --- | --- | --- | --- | --- | --- |
| **Skin** | + (2/3) | + (1/3) | ++ (2/3) | + (3/3) | + (1/3) | + (3/3) | + (1/3) | + (1/3) | Follicular epithelial cells, keratinocytes, endothelial and inflammatory cells | Mixed inflammatory cell infiltrate in dermis, oedema, congestion, microthrombi, vasculitis. |
| **Pectoral muscle** | + (2/3) | + (1/3) | + (2/3) | + (3/3) | + (2/3) | + (3/3) | + (1/3) | + (1/3) | Myocytes | Focal degenerated and necrotic fibers with mild inflammatory infiltrate. |
| **Nasal cavity** | ++ (3/3) | + (1/3) | ++ (2/3) | ++ (3/3) | ++ (2/3) | ++ (3/3) | + (1/3) | ++ (1/3) | Respiratory and olfactory cells, Bowman glands. Nasal glands, nasolacrimal duct, endothelial and inflammatory cells | Areas of necrosis of epithelial cells (loss of continuity, loss of cilia) with mixed inflammatory cell infiltration, congestion. |
| **Lung** | +++ (2/3) | ++ (1/3) | +++ (2/3) | +++ (3/3) | ++ (2/3) | ++ (3/3) | ++ (1/3) | ++ (1/3) | Air capillaries cells, endothelial and inflammatory cells | Increase of cellularity (mixed inflammatory cells) in air capillaries interstitium, focal areas of necrosis in pneumocytes, microthrombi, intersticial oedema. |
| **Central nervous system** | ++ (3/3) | + (1/3) | +++ (2/3) | ++ (3/3) | ++ (2/3) | ++ (3/3) | + (1/3) | + (1/3) | Neurons, glial cells, Purkinje cells (cerebellum), endothelial cells | Areas of necrosis (chromatolysis and spongiosis) with microgliosis, congestion. |
| **Pancreas** | ++ (3/3) | ++ (1/3) | ++ (2/3) | ++ (3/3) | + (1/3) | ++ (3/3) | + (1/3) | + (1/3) | Exocrine acinar cells, endothelial and inflammatory cells | Areas of necrosis with inflammatory cell infiltration. |
| **Heart** | +++ (2/3) | +++ (1/3) | +++ (2/3) | ++ (3/3) | ++ (2/3) | ++ (3/3) | ++ (1/3) | + (1/3) | Myocardiocytes, endothelial and inflammatory cells | Areas of necrosis of myocardiocytes (fiber degeneration and hyalinization) with mixed inflammatory cell infiltrate. |
| **Spleen** | ++ (2/3) | ++ (1/3) | ++ (2/3) | ++ (3/3) | ++ (2/3) | ++ (3/3) | ++ (2/3) | + (2/3) | Endothelial and inflammatory cells | Areas of necrosis (including germinal centers) with mixed inflammatory infiltrate. |
| **Thymus** | ++ (2/3) | ++ (1/3) | ++ (2/3) | ++ (3/3) | ++ (2/3) | ++ (2/3) | ++ (1/3) | + (1/3) | Endothelial and inflammatory cells | Areas of necrosis with inflammatory cell infiltrate in medulla and more restricted inflammatory cell infiltrate in cortex, congestion. |
| **Liver** | + (2/3) | ++ (1/3) | ++ (2/3) | + (3/3) | + (2/3) | ++ (3/3) | ++ (1/3) | + (1/3) | Hepatocytes, Kuppfer cells, endothelial and inflammatory cells | Focal areas of necrosis with mixed inflammatory cell infiltrate, mild distension of hepatic sinusoids, congestion. |
| **Kidney** | + (2/3) | + (1/3) | + (2/3) | + (3/3) | + (2/3) | + (3/3) | + (1/3) | + (1/3) | Epithelial tubular cells, endothelial and inflammatory cells | Acute tubular necrosis with mild mixed inflammatory infiltrate, congestion. |
| **Proventriculus** | + (2/3) | ++ (1/3) | ++ (2/3) | + (2/3) | + (2/3) | + (3/3) | + (1/3) | + (1/3) | Epithelial cells of the proventricular glands, endothelial and inflammatory cells | Focal areas of necrosis in glandular cells and mainly in lymphoid tissue. |
| **Gizzard** | ++ (2/3) | ++ (1/3) | ++ (2/3) | ++ (3/3) | + (2/3) | ++ (3/3) | ++ (1/3) | + (1/3) | Epithelial cells of the ventricular glands, smooth muscle cells, endothelial and inflammatory cells | Areas of necrosis in glandular cells with mixed inflammatory cell infiltration, muscular cells necrosis and degeneration. |
| **B.Fabricius** | + (2/3) | + (1/3) | ++ (2/3) | + (3/3) | + (2/3) | ++ (3/3) | + (1/3) | + (1/3) | Endothelial and inflammatory cells | Areas of necrosis with inflammatory cell infiltrate (macrophages and heterophils) in germinal centers and interstitial compartment. |
| **Small intestine** | + (2/3) | + (1/3) | + (2/3) | + (3/3) | + (2/3) | ++ (3/3) | + (1/3) | + (1/3) | Endothelial and inflammatory cells | Necrosis of lymphoid tissue with mixed inflammatory infiltrate. |
| **Large intestine** | + (2/3) | + (1/3) | ++ (2/3) | + (3/3) | + (1/3) | + (3/3) | - | + (1/3) | Endothelial and inflammatory cells | Necrosis of lymphoid tissue with mixed inflammatory infiltrate. |
